# LIFETIME CLOSE TO THE END: EFFECTS ON PERCEIVED TIME AND AGING IN OLDER ADULTS WITH ADVANCED CANCER

**DOI:** 10.1093/geroni/igac059.1675

**Published:** 2022-12-20

**Authors:** Hans-Werner Wahl, Katsiaryna Laryionava, Anton Schönstein, Pia Heussner, Wolfgang Hiddemann, Eva Winkler

**Affiliations:** Universität Heidelberg, Heidelberg, Baden-Wurttemberg, Germany; Heidelberg University, Heidelberg, Baden-Wurttemberg, Germany; Heidelberg University, Heidelberg, Baden-Wurttemberg, Germany; Klinikum Garmisch-Partenkirchen GmbH, Garmisch-Partenkirchen, Bayern, Germany; Ludwig-Maximilians University, Munich, Baden-Wurttemberg, Germany; Heidelberg University, Heidelberg, Baden-Wurttemberg, Germany

## Abstract

This study addressed two questions: (1) Does advanced cancer in later life affect a person’s awareness of time and their subjective age? (2) Are awareness of time and subjective age associated with distress, perceived quality of life, and depression? We assessed patients with terminal cancer (OAC, n = 91) and older adults with no life-threatening disease (OA, n = 89). All participants were age 50 or older. OAC perceived time as being a more finite resource and felt significantly older than OA controls. Feeling younger was significantly related with better quality of life and lower levels of distress. In the OA group, feeling younger was also associated with reduced depression. Perceiving time as a finite resource was related to higher quality of life in the OA group. Indicators of an older person’s awareness of time and subjective aging differ between those with advanced cancer versus controls without a terminal disease.

